# Short-Term Repeated Transcutaneous Spinal Cord Stimulation Yields Sustained Orthostatic Benefits in Chronic Cervical SCI: A Case Study

**DOI:** 10.3390/jcm14196700

**Published:** 2025-09-23

**Authors:** Einat Engel-Haber, Akhil Bheemreddy, Mehmed Bugrahan Bayram, Manikandan Ravi, Brittany Snider, Steven Kirshblum, Gail F. Forrest

**Affiliations:** 1 Kessler Foundation, West Orange, NJ 07052, USA; 2 Department of Physical Medicine and Rehabilitation, Rutgers New Jersey Medical School, Newark, NJ 07103, USA; 3 Kessler Institute for Rehabilitation, West Orange, NJ 07052, USA

**Keywords:** spinal cord injury, spinal cord transcutaneous stimulation, spinal cord stimulation, blood pressure, cardiovascular, orthostatic hypotension, adaptation

## Abstract

**Background/Objectives:** Cardiovascular (CV) dysfunction and, specifically, orthostatic hypotension, may significantly impact the quality of life of individuals with spinal cord injuries (SCIs) at T6 or above. While spinal cord transcutaneous stimulation (scTS) has shown immediate effects on blood pressure regulation, its long-term effects remain largely unexplored. **Methods:** This case study examines the sustained effects of scTS on blood pressure regulation and orthostatic tolerance in a 33-year-old female with cervical (C4) complete SCI sustained two years earlier. This individual underwent an initial baseline tilt test without stimulation, completed six 30 min scTS-CV sessions (cardiovascular-focused stimulation) over two weeks as the “training” phase, and then had repeated tilt tests without stimulation posttraining. **Results:** Following training, the participant demonstrated an improvement in orthostatic tolerance, maintaining a 70° tilt for 30 min, compared to only 3 min at baseline, in a tilt test (without stimulation) conducted one day posttraining. Self-reported reduction in orthostatic burden and decreased midodrine dependence were also observed for several weeks, with improvements diminishing by 6 weeks posttraining. **Conclusions:** These observations suggest that brief, repeated scTS-CV sessions may lead to sustained improvements in orthostatic tolerance beyond the immediate period of stimulation. Although the duration of these effects has yet to be established, this approach could offer a non-invasive alternative for managing CV dysfunction in SCIs.

## 1. Introduction

Cardiovascular (CV) dysfunction represents a critical challenge for individuals with spinal cord injuries (SCIs), especially those with cervical or high thoracic (≥T6) injuries. These individuals experience complex autonomic instability characterized by unstable low blood pressure (BP), orthostatic hypotension (OH), autonomic dysreflexia (AD), and various physiological challenges.

OH, defined by a 20/10 mmHg drop in systolic/diastolic blood pressure when transitioning from supine to an upright position, typically emerges soon after injury and often persists throughout life [1]. While symptoms may diminish over time, OH typically remains clinically detectable. The condition manifests through various symptoms including dizziness, blurred vision, weakness, fatigue, nausea, headache, palpitations, and syncope [2,3,4,5]. Daily BP fluctuations combined with chronic hypotension correlate with reduced cerebral blood flow velocity and cognitive deficits [6,7]. Moreover, BP instability significantly impairs daily functioning and quality of life and likely contributes to the 3- to 4-fold elevated risk of stroke and heart disease observed in this population [8,9,10,11].

The current treatment landscape for individuals with low BP and OH is notably limited. Existing pharmacological (e.g., midodrine, droxidopa, and fludrocortisone) and non-pharmacological (e.g., salt and fluid regulation, abdominal binders, and elastic stockings) interventions demonstrate inconsistent efficacy, with possible side effects and dosage restrictions that compromise long-term management [5,12,13]. Recent research underscores the potential of spinal cord stimulation—both epidural and transcutaneous—in regulating BP in individuals with SCI [14]. Studies highlight its versatility in both preventing and mitigating AD [15,16], an elevation in BP triggered by noxious or non-noxious stimuli below the level of injury, and increasing BP to alleviate OH [17,18,19,20,21].

However, a critical gap remains: the long-term hemodynamic effects of stimulation are not well understood. Existing data from epidural studies suggest a sustained effect, as ‘training’, i.e., a series of spinal cord epidural stimulation sessions focusing on cardiovascular function (scES-CV), in individuals with chronic cervical SCI, resulted in improved BP response and increased tolerance to orthostatic challenges [22,23,24]. Two studies included approximately 90 scES-CV training sessions each, and in post-training tilt tests, participants’ BP remained stable even without stimulation, whereas before training, they experienced significant BP drops [22,24]. Another study incorporated stimulation training for 15 weeks (approximately three sessions per week) and found that following training, lower stimulation amplitude was needed to prevent systolic BP (SBP) drops during orthostatic challenges [23]. However, drawbacks of these studies include the number of sessions required and the invasive nature of scES. Moreover, although these and other studies report that participants experienced a reduction in the autonomic burden and improvements in daily activities and community participation during—and possibly shortly after—the stimulation period, it remains unclear whether these benefits were sustained after the study ended and for how long [19,23,24,25].

In contrast to scES, spinal cord transcutaneous stimulation (scTS) offers a non-invasive alternative, with initial studies demonstrating immediate potential benefits in raising BP [26] and reducing OH [18,21]. However, current research in this area remains limited, and the long-term effects of scTS-CV training (repeated scTS sessions focusing on cardiovascular function) have yet to be investigated. This study provides preliminary evidence based on one individual with chronic cervical SCI, suggesting that a short course of repeated scTS-CV sessions was associated with cardiovascular adaptations sustained beyond the immediate training period. This underscores the need for more comprehensive future research in this promising field, to better understand the mechanisms underlying this autonomic neuroplasticity and to explore the broader therapeutic potential of this approach.

## 2. Materials and Methods

### 2.1. Case Description

This case study involved a 33-year-old female (height: 5′4″, weight: 100 lbs.) with a chronic, complete cervical SCI sustained 2 years prior to the study as the result of a motor vehicle crash. She had not previously undergone any spinal cord stimulation before enrolling in this study. Comprehensive examination performed according to the International Standards for Neurological Classification of SCI (ISNCSCI) [27] revealed a C4 American Spinal Injury Association (ASIA) Impairment Scale (AIS) injury, with bilateral motor levels at C7. Her medication regimen included midodrine, oxybutynin, and tizanidine. The participant reported persistent low BP, episodic OH, and occasional AD. She noted that prior to the injury, her BP was in the normal–lower range, typically between 110 and 120 mmHg for SBP.

Following the SCI, her OH manifested in symptoms such as dizziness, temporary vision, and hearing loss and, eventually, loss of consciousness. To prevent these, she takes midodrine (10 mg) every 4 h (typically at 8 a.m., noon, 4 p.m., and 8 p.m.) but increases the frequency to every 3 h during social events. She occasionally experiences syncope, particularly when transitioning from lying down to sitting up. At home, she reports having faced several alarming incidents, including rapid BP drops while leaning forward in her wheelchair, which has caused disorientation and, in some cases, led her to fall out of the chair. In one notable instance, a sudden drop in BP caused her to accidentally drive the power wheelchair into a door frame, breaking part of the trim. Additionally, she experiences mild episodes of AD, typically presenting as blotchy skin and goosebumps, which are generally resolved through bladder catheterization.

### 2.2. Study Design and Assessment Procedures

The participant was enrolled in a clinical trial (NCT05725499) investigating the effects of scTS on BP regulation in five individuals with chronic SCI and OH. The study protocol was approved by the local Institutional Review Board (IRB). The trial included two primary aims, which were not the focus of this manuscript and will be reported separately: (1) to assess a novel CV stimulation mapping technique, and (2) to determine the immediate effect of stimulation on BP during an orthostatic provocation. In addition, the study included an exploratory aim, which is the focus of this case report—to evaluate whether improvements in BP response were sustained following a short course of repeated scTS CV sessions (“training”).

At the time of analysis, three participants had completed the study protocol for the primary aims. However, only one participant completed the exploratory training protocol in full and is described in this case report. Of the other two participants, one did not initiate training due to medical complications, and the other experienced a prolonged interruption in training (both unrelated to the study), precluding their inclusion in this exploratory analysis.

The general protocol was as follows: This investigation began with a 70° baseline tilt test to document OH severity. Stimulation mapping (Aim 1) sessions were conducted to identify effective BP-elevating scTS configurations (i.e., ‘scTS-CV’) that did not elicit substantial lower extremity muscle contractions. Following mapping, a tilt test was conducted to assess the immediate effect of stimulation (Aim 2). To assess longer-term effects of stimulation (Exploratory aim), the participants completed six 30 min scTS-CV sessions (‘training’) while seated in their wheelchair over a two-week period, followed by a post-training tilt test. As noted earlier, this case report describes the participant who completed the training protocol in its entirety. Based on the notable outcome of the post-training test, the follow-up period for this case was extended with IRB approval, and a final tilt test was performed ~6 weeks after training completion.

#### 2.2.1. Tilt Testing

An established procedure to assess OH [24,28] was conducted using a Hi–Lo tilt table (Metron T8610, Performance Health Supply, Inc., Cedarburg, WI, USA). For safety, the participant was secured to the bed at the tibial tuberosity, iliac crest, and below the axilla, with feet anchored against the footplate. The protocol began with 10 min in a supine position, allowing time for a BP device calibration and baseline CV assessment. The transition to the 70° position was attempted within 2 min, with brief stops at intermediate angles (e.g., 30°, 45°, 60°) to ensure participant comfort. Continuous hemodynamic measurements were recorded throughout the tilt, and orthostatic symptoms were assessed every 5 min or sooner if symptoms intensified, using a scale from 0 (no impact) to 10 (most severe) for headaches, dizziness, blurred vision, nausea, weakness, confusion, fatigue, ringing in the ears, and feeling of passing out [29]. Participants remained at the 70° angle for 30 min, unless orthostatic symptoms or a severe drop in BP necessitated early termination or adjustment to a lower tilt angle. Additionally, our team monitored physiological signs such as spontaneous yawning and facial pallor, which served as early warnings of cerebral hypoperfusion and indicated that we were approaching the point at which the tilt should be terminated. While BP thresholds (i.e., the BP range in which participants remain comfortable and symptom-free) vary individually, particularly in those with high SCI who often tolerate lower BP, tilt was generally discontinued if SBP fell below 60–65 mmHg. The session concluded with a final 10 min supine period.

#### 2.2.2. Cardiovascular Monitoring and Analysis

Throughout the different study sessions, continuous hemodynamic measurements (SBP, diastolic BP [DBP], and heart rate [HR]) were recorded using the Finapres non-invasive BP system through a single finger cuff (Finapres Medical Systems, Amsterdam, The Netherlands) with height correction to compensate for vertical hand movement relative to heart level [18,30,31]. Data were sampled at 1 kHz using the ADInstruments Powerlab 16/35 data acquisition system (ADInstruments, Dunedin, New Zealand). Brachial BP measurements (Welch Allyn, Monitor 6000 Series, Skaneateles Falls, NY, USA) were collected at 1-min intervals on the contralateral side to the finger cuff. Offline calibration of finger-to-brachial BP was performed as detailed in our previous manuscript [21].

#### 2.2.3. Stimulation Mapping

Several sessions were performed to identify configurations (‘scTS-CV’) that increased and stabilized SBP within the normotensive range (110–120 mmHg) [17]. A five-channel constant current stimulator (NeoStim-5, Cosyma Inc., Moscow, Russia) delivered sub-threshold scTS through round 2.5 cm self-adhesive electrodes placed as cathodes over the dorsal skin at the midline of spinous processes in predetermined locations, including C5/6, T7/8, T11/12, L1/2, and S1/2 [18,21]. Rectangular anode electrodes (8 × 13 cm) were placed over the anterior iliac crests. Stimulation was administered at various frequencies (primarily 2 and 30 Hz) using rectangular pulses of 1 ms duration (or less), filled with a 5 kHz carrier frequency. The intensity was progressively increased in 5 mA increments, up to a maximum of 150 mA, based on tolerance and with the goal of increasing SBP to the normotensive range.

During mapping sessions, continuous real-time BP measurements were recorded and synchronized using finger plethysmography, brachial BP, and surface electromyography (sEMG) data. The sEMG data were collected at a frequency of 10 kHz using 16-channel MA400 systems (Motion Lab Systems, Baton Rouge, LA, USA) to ensure BP elevations were not caused by lower extremity muscle contractions. Bipolar sEMG electrodes were placed bilaterally on the gluteus maximus, biceps femoris, rectus femoris, vastus lateralis, medial gastrocnemius, soleus, and tibialis anterior muscles [32]. Stimulation artifacts were removed prior to EMG analysis [33,34]. Lower extremity motor activity was further assessed through direct observation and participant feedback regarding sensations of spasms. The mapping procedure is also discussed in our previous study [21].

#### 2.2.4. Training

A regimen consisting of six 30-min sessions of scTS-CV, using stimulation parameters obtained during mapping, while the participant was in their wheelchair, was conducted over a period of approximately two weeks. During the sessions, continuous hemodynamic measures were collected. Participants completed an Autonomic Dysfunction Following Spinal Cord Injury (ADFSCI) questionnaire twice, once for pre-training settings and another after the final training session, to evaluate changes in orthostatic burden experienced in daily life [29].

## 3. Results

The participant was instructed to avoid caffeine and alcohol for at least 12 h before each session and to empty her bladder before testing. She refrained from taking midodrine 6 h prior to each session. Each session began with safety screening using an in-house form to identify any events that might make testing unsafe or its results unreliable. A clinician was present at every session to monitor for adverse events, skin changes, participant discomfort, and other safety concerns. No study-related adverse events occurred, aside from expected redness at the stimulation and EMG electrode sites in this participant with highly sensitive skin. At routine pre-session assessments, seated SBP with an abdominal binder ranged from 65 to 124 mmHg (mean: 95 ± 14 mmHg). Without the binder, seated SBP averaged 87 ± 15 mmHg.

Based on the mapping sessions, T7/8 and L1/2 were chosen for electrode placement for the subsequent training sessions, as stimulation at these levels successfully raised the participant’s BP while minimizing lower extremity activation. These selections (‘scTS-CV’) were used in combination during the 30 min seated training sessions, with L1/2 amplitudes reaching up to 150 mA and T7/8 to 80 mA. The stimulation was delivered at a frequency of 30 Hz, using a monophasic waveform with a pulse width of 1 ms and a carrier frequency of 5 kHz.

**Figure 1:**
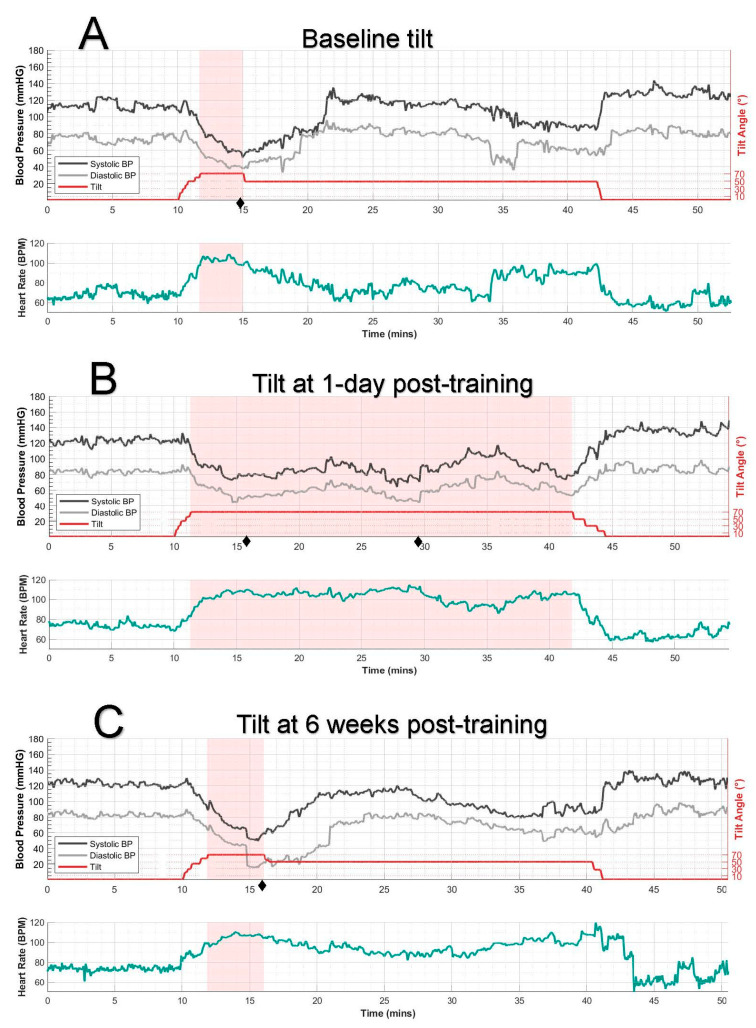
A series of 70° tilt tests (without stimulation) showing systolic (SBP) and diastolic blood pressure (DBP), tilt angle (top plots), and heart rate (bottom of each plot). The pink shaded region marks the duration the participant sustained the 70° tilt position. Symptoms are denoted by ♦ on the timeline and described below. (**A**) Pre-training: The participant maintained the 70° tilt for only 3 min before SBP dropped to 60 mmHg, accompanied by orthostatic symptoms (dizziness 4/10, blurred vision 4/10, passing out sensation 5/10♦). The tilt angle was then reduced to 50°, which was better tolerated. (**B**) A tilt test conducted one day after the final training session (having completed 6 sessions in two weeks) showed significant improvement in BP, although still not reaching the normotensive range. SBP remained primarily above ~80 mmHg. Mild symptoms occurred at 6 min (fatigue 2/10♦, other symptoms 0/10) and 20 min (fatigue 3/10, passing out sensation 1/10♦) into the tilt but resolved spontaneously; no other symptoms were reported during the testing period, with the participant completing the full 30-min tilt. (**C**) Six weeks posttraining: the participant’s BP response reverted to baseline levels. After 4 min at 70°, low SBP and symptoms (dizziness 3/10, blurred vision 2/10, fatigue 4/10, passing out sensation 3/10♦) necessitated reducing the tilt angle to 50°.

The participant completed three tilt tests: before training, one day after training, and 6 weeks posttraining. One day after completing the six-session stimulation training protocol, the participant exhibited a marked improvement in orthostatic tolerance compared to baseline (see Figure 1): Initial assessments had indicated severe orthostatic intolerance, characterized by an inability to sustain the 70° tilt position beyond three minutes, increased severity of orthostatic symptoms, and a drop in SBP to 60 mmHg. In contrast, the 1-day post-training evaluation showed substantial progress—the participant successfully maintained the full 30-min duration at 70° tilt, experienced a substantial reduction in symptoms, and demonstrated improved SBP stability, with levels only dropping to 75–80 mmHg (and often higher) during the tilt. In a repeated tilt test conducted 6 weeks after training, these improvements had diminished.

Of note, as depicted in Table 1, although there remained a notable drop in SBP (Δ = −36 mmHg) during the tilt conducted 1 day following training (Figure 1B), it was still a smaller drop compared to baseline and 6-week post-training tilt tests (Δ = −43 and −52, respectively). Moreover, the average SBP during the 70° tilt at 1-day post-training was significantly higher (88 mmHg) compared to the baseline and 6 weeks post-training tilt tests (both of which were 69 mmHg). The diminished drop in SBP at the 1-day post-training timepoint likely explains the marked reduction in symptoms and ability to sustain the 70° position compared to the tilt tests at the baseline and 6-week timepoints. Of note, supine baseline SBP before each tilt ranged from 112 to 124 mmHg, reflecting expected variability in the SCI population.

Self-reported changes in orthostatic burden: Towards the end of the training period and for several weeks following training, the participant reported improvement in her orthostatic burden at home. She stated, “I’ve noticed I don’t need midodrine as frequently as before,” and “My blood pressure doesn’t drop as sharply as it used to.” Notably, there were instances in which she forgot to take her medication, something that had not occurred prior to training. In the ADFSCI questionnaire, her reported standing/sitting time tolerance improved from the highest severity (4/4) pre-training (“On most occasions, I can stand/sit less than 1 min before experiencing orthostatic symptoms”) to a lower severity (2/4) posttraining (“On most occasions, I can stand/sit 5–14 min before experiencing orthostatic symptoms”). Other ADFSCI-reported OH measures, as well as AD frequency and severity, remained unchanged. This effect gradually waned and, at the 6-week follow-up, the participant reported feeling her orthostatic burden had returned to baseline.

## 4. Discussion

Multiple prior studies have established the immediate impact of stimulation, particularly epidural stimulation, on CV responses, demonstrating its ability to ameliorate orthostasis during a tilt test. Nonetheless, the enduring nature of these effects over time remains an open question. This report provides preliminary evidence from one individual that a brief course of stimulation sessions was associated with prolonged stabilization of blood pressure and symptom relief beyond the immediate stimulation session. This finding is particularly notable as it questions the assumption that prolonged or intensive stimulation sessions are required to achieve training effects while also suggesting that non-invasive transcutaneous stimulation could be sufficient, as opposed to invasive epidural methods [22,23,24].

Previous epidural stimulation studies have demonstrated improvements in orthostatic tolerance in tilt tests following training, but typically after substantially longer intervention periods. Harkema et al. [22] reported that four individuals with chronic SCI who underwent approximately 89 two-hour daily sessions of scES-CV showed stabilized BP during orthostatic challenges, even without stimulation, following training. Similarly, Bloom et al. [24] described a case where an individual with chronic motor complete SCI completed 97 scES-CV sessions, after which a 70° tilt-up test without stimulation revealed increased BP, enhanced cerebral blood flow, and improved orthostatic tolerance compared to pre-training assessments. Another recent case report by Gorgey et al. [23] involved an individual with a chronic motor complete cervical SCI. Percutaneous scES in the T11–L1 vertebrae was applied. Although initially configured for an exoskeleton study, after four weeks of training (3 sessions per week), the participant self-reported reduced symptoms of low BP when using this configuration outside of training in daily life. Subsequently, 45° tilt table tests, both without and with stimulation, were performed initially and after 15 weeks of additional stimulation sessions. After 15 weeks, a lower stimulation amplitude was necessary to prevent a drop in SBP upon orthostatic challenge.

Our preliminary observations in one participant show that after six 30 min scTS-CV sessions (2–3 times weekly), orthostatic provocation tests without stimulation revealed better tolerance than at baseline. These case-based findings underscore the need for further research into the minimal effective dose and the persistence of these effects over time. However, caution is needed because day-to-day variability in baseline supine SBP among individuals with SCI can influence symptom severity. On days with lower resting SBP, modest pressure drops may trigger pronounced symptoms, whereas higher baselines may better absorb larger declines. Notably, despite the overall improvement one day after training, the participant still experienced a fall in SBP during tilt testing. Moreover, given that the inter-day reliability of tilt tests has not been established, we cannot exclude the possibility that some of the day-to-day variability observed reflects measurement fluctuations rather than true physiological change.

In addition to quantitative effects measured during tilt testing, there are reports from multiple scES studies [19,23,24,25], where participants have reported general reductions in orthostatic burden (one report even abolishing the need to take midodrine [19]), increased vitality, and improved quality of life during study participation. In a comprehensive study by Boakye et al. [25] involving 25 participants, individuals reported transformative experiences: “(Previously) passing out, didn’t want to be around people, couldn’t engage, now can sit with people all day”, “Not dizzy, heart not skipping a beat, not passing out,” “Conversations for a long period of time”, “I can stay out longer”, and “Do more things with my family”. These testimonials align with some of the improvements reported by our participant, who required midodrine less frequently than usual and felt that her BP was more stable. This observation was further supported by her responses to the ADFSCI questionnaire, indicating significant clinical benefits that extend beyond measurable physiological outcomes. In our study, however, when reassessment occurred 6 weeks after training, these clinical benefits were no longer evident.

Of note, seated mapping sessions aim to stabilize SBP within 110–120 mmHg, with the rationale being that this range aligns with the healthy SBP standards set by the American College of Cardiology [35,36]. However, during a 70° tilt, this range may not be an appropriate goal for individuals with SCI and chronically low baseline BP, especially if these individuals had relatively low pre-injury BP. Despite only achieving an average SBP below 90 mmHg during the 70° tilt test conducted one day after training, this tilt test is still considered a clinical short-term success as this individual hardly experienced any orthostatic symptoms (only a brief episode of OH symptoms 20 min into the tilt that resolved spontaneously) and successfully completed the full 30-min 70° tilt protocol. Functional tolerance and symptom management may be additional meaningful clinical endpoints.

Lau et al. [37] reported a case of an individual with a sub-acute cervical complete SCI in which stimulation began three months post-injury for refractory severe OH that limited sitting to under 30 min. Across nine sessions over three weeks, the participant underwent 30 min of seated stimulation at the T7/8 and T12/L1 levels. Despite using different settings from ours—pulse width 450 μs vs. 1000 μs, frequency 100 Hz vs. 30 Hz, and no carrier frequency vs. 5 KHz—stimulation was able to sustain a full 30-min sit. In addition, and most relevant to this discussion, after these nine sessions, the authors reported that upright sitting tolerance doubled to 60 min (without stimulation), markedly improving the quality of life for this individual. Upon discharge, the participant was advised to continue the prescribed daily stimulation regimen at home. This case reinforces our key findings that a brief scTS course can deliver longer-lasting cardiovascular benefits. A key distinction is that our participant was in the chronic stage of SCI, when natural recovery is minimal, whereas theirs was in the sub-acute phase, making it more difficult to separate stimulation effects from spontaneous recovery.

Considering CV dysfunction at both extremes—OH and AD—a key question is whether training-induced BP adaptation also improves AD episodes, given its reported immediate preventive and mitigating effects on AD [15,16]. Although not quantitatively evaluated in this study, and the participant did not notice a change in frequency or severity of AD (as noted by the ADFSCI questionnaire), prior studies suggest that it does. In humans, DiMarco et al. [38,39] reported that multi-week epidural/percutaneous stimulation initially triggered asymptomatic BP surges in three AD-susceptible participants, but these responses waned over daily sessions until stimulation no longer evoked significant hemodynamic changes after several weeks. Animal models provide stronger support. In rats, scTS prevented colorectal distension-evoked AD and, when applied as a six-week multisession therapy post-SCI (daily or alternate days), significantly reduced AD severity even without concurrent stimulation for at least one week after treatment ended [16]. Richardson et al.’s [40] 1979 study also reported resolution of AD episodes following epidural neurostimulation alongside improvements in spasticity and bladder function, although this may reflect indirect effects from eliminating triggering factors rather than direct neuromodulation.

While the mechanisms mediating the immediate BP response, believed to involve sensory afferent fibers activating spinal interneurons and sympathetic preganglionic neurons (SPNs), are relatively well-characterized [41], there is an ongoing debate as to the pathophysiology of the BP increase. Some propose it reflects a direct neuromodulatory effect, while others argue it represents an AD response [21]. Although this topic is not addressed in detail here, the sustained increase in BP after training, even in the absence of active stimulation, suggests it may not be attributable solely to AD.

The mechanisms driving the sustained BP response remain poorly understood and sparsely documented in the literature. A full exploration of the neuroplastic mechanisms behind these adaptations is beyond the scope of this manuscript; however, they likely reflect a combination of processes operating on different timescales. Short-term neural adaptations, occurring over days to weeks, are the most plausible drivers in our two-week stimulation protocol. Stimulation may enhance baroreflex sensitivity, increase excitability of spinal sympathetic circuits, and strengthen synaptic efficacy within autonomic pathways by reflex conditioning [22,42]. In contrast, true vascular remodeling may require a longer duration of exposure to the intervention, possibly several months. This includes improvements in endothelial function, reductions in arterial stiffness, and structural changes in the microvasculature. Likewise, myelin preservation, remyelination, and adaptive axonal sprouting around the lesion develop more slowly [41]. These hypotheses remain untested; future studies should incorporate direct measures of baroreflex gain, sympathetic nerve activity, and vascular biomarkers to delineate the relative contributions and time courses of the various adaptations.

As a case report with a single participant who completed the exploratory training protocol, our findings have limited generalizability and should be interpreted as preliminary and hypothesis generating. In this unblinded design, non-specific factors such as placebo effects, regression to the mean, recall bias, and observer-expectancy effects may have driven some of the observed improvements, particularly those based on self-report. However, they provide compelling preliminary evidence that warrants further investigation in larger, controlled studies employing rigorous blinding, randomization, and objective autonomic assessments to confirm true treatment effects.

Future studies should investigate the impact of training using scTS on both OH and AD, the latter by incorporating provocations such as urodynamic testing or digital anorectal stimulation (DARS). They should systematically optimize stimulation parameters, session duration, and training frequency to define dose–response relationships and sustain BP adaptations. Critically, these investigations must compare extended initial training protocols with periodic “booster” sessions, since the orthostatic improvements observed here waned by six weeks posttraining, indicating that a brief training course is unlikely to provide durable clinical benefit. Finally, additional objective measures such as 24 h ambulatory blood pressure monitoring (ABPM) should be adopted to quantify the frequency and severity of both hypotensive and hypertensive episodes before and after training, thereby capturing daily BP variability and the intervention’s effects.

## 5. Conclusions

As a preliminary, single-participant, hypothesis-generating observation, our findings suggest that a concise regimen of transcutaneous spinal stimulation, far fewer sessions than previously reported, correlated with short-term sustained alterations in cardiovascular responses, suggesting further study of minimal effective protocols. Achieving sustained BP improvement with non-invasive transcutaneous spinal stimulation training underscores the need to explore this approach as a non-surgical therapeutic option and its impact on cardiovascular outcomes.

## Figures and Tables

**Table 1:** BP response during a 70° tilt (without stimulation) before, 1 day after, and 6 weeks posttraining.

Tilt	Duration of 70° Tilt (min)	SBP, Avg ± SD		DBP, Avg ± SD		HR, Avg ± SD		Max Symptoms Total
		Supine	70° Tilt	Supine	70° Tilt	Supine	70° Tilt	
Baseline	3.3	112 ± 4	69 ± 10	74 ± 4	46 ± 6	68 ± 5	102 ± 7	13
		Δ = −43		Δ = −28		Δ = +34		
1 day posttraining	30.5	124 ± 3	88 ± 11	84 ± 2	61 ± 9	74 ± 3	100 ± 11	4 (0) *
		Δ = −36		Δ = −23		Δ = +26		
6 weeks posttraining	4.2	121 ± 3	69 ± 14	82 ± 3	41 ± 17	74 ± 4	105 ± 4	12
		Δ = −52		Δ = −41		Δ = +31		

BP, blood pressure; SBP, systolic blood pressure; DBP, diastolic blood pressure; HR, heart rate. * After 20 min in the 70° tilted position, the participant reported a fatigue level of 3/10 and a 1/10 sensation of feeling faint. These symptoms resolved spontaneously, and she completed the 30-min test without further issues; SBP, systolic blood pressure; DBP, diastolic blood pressure; HR, heart rate; avg, average.

## Data Availability

The data that support the findings of this study are available by contacting the corresponding author via e-mail.

## Abbreviations

The following abbreviations are used in this manuscript:

AD	Autonomic dysreflexia
ADFSCI	Autonomic Dysfunction Following Spinal Cord Injury
BP	Blood pressure
AIS	American Spinal Injury Association (ASIA) Impairment Scale
CV	Cardiovascular
DBP	Diastolic blood pressure
HR	Heart rate
ISNCSCI	International Standards for Neurological Classification of SCI
OH	Orthostatic Hypotension
SBP	Systolic blood pressure
SCI	Spinal cord injury
ScES	Spinal cord epidural stimulation
ScES-CV	Spinal cord epidural stimulation focusing on cardiovascular function
ScTS	Spinal cord transcutaneous stimulation
ScTS-CV	Spinal cord transcutaneous stimulation focusing on cardiovascular function
sEMG	Surface electromyography
